# Supplementary material for: pCR and 2-Year Disease-Free Survival: A Combination of the Two Endpoints as a New Classification for Locally Advanced Rectal Cancer Patients—An Updated Pooled Analysis of Eleven International Randomized Trials
Source: Cancers (Basel). 2023 Jun 16;15(12):3209. doi: 10.3390/cancers15123209 (PMC10295980; doi:10.3390/cancers15123209)
Supplement: Supplementary file 1 [file cancers-15-03209-s001.zip › cancers-2400527-supplementary.pdf]

Supplemental material

Table S1. Randomized phase III clinical studies included in the analysis.

Study, first author (year)	Design	Inclusion criteria	Accrual time (years)	N° Study patients	N° Included patients (%)	pCR rate	Clinical endpoint	Main findings
CAO/ARO/AIO-94, Sauer R [12] (2004)	NAD-CRT (50Gy + 5-FU first and last week) vs ADJ-CRT (50Gy + 5-FU first and last week)	Stage II-III<br>Age <75<br>Tumor located within 15 cm from anal margin<br>No previous pelvic RT and/or CT<br>No previous history of cancer	1995- 2002	799	481	NAD-CRT 8%	OS	NAD-CRT:<br>- improves LC;<br>- reduces toxicity;<br>- does not improve OS
Polish I, Bujko K [14] (2006)	SC-RT (25 Gy) Vs NAD-CRT (50.4 Gy + 5-FU first and last week) +/- ADJ-CT	cT3-T4 resectable cancer<br>No sphincter involvement on DRE<br>Lower tumor margin accessible to DRE	1999-2002	312	129	SC-RT 0.7%<br>NAD-CRT 16.1%	OS, DFS, LR, late toxicity	NAD-CRT<br>- does not improve survival, LC and toxicity<br>- increases complete tumor response, R0 surgery rates
EORTC 22921, Bosset JF [16] (2006)	nRT (45 Gy) vs NAD-CRT (45 Gy + 5-FU first and last week) vs nRT (45 Gy) + ADJ-CT (4 cycles) vs NAD-CRT (45 Gy + 5FU first and last week) + ADJ-CT (4 cycles)	T3 or resectable T4 M0<br>ECOG PS 0 – 1<br>Age < 80<br>Tumor located within 15 cm from anal margin	1993-2003	1011	748	nRT 5.3%<br>NAD-CRT 13.7%	OS	NAD and ADJ-CRT:<br>- improve LC;<br>- do not improve OS
FFCD 9203, Gerard GP [17] (2006)	nRT (45Gy) vs NAD-CRT (45Gy + 5-FU first and last week)	T3 or resectable T4 M0<br>ECOG PS 0 – 1<br>Age <75<br>No previous pelvic RT	1993- 2003	742	529	nRT 3.6%<br>NAD-CRT 11.4%	OS	NAD-CRT:<br>- significantly improves LC;<br>- has no impact on OS;<br>- moderately increases acute toxicity;<br>- is recommended for T3-4, N0-2, M0 adenocarcinoma of middle and distal rectum.
TROG 01.04, Ngan SY [18] (2012)	SC-RT (25Gy) Vs NAD-CRT (50.4 Gy + 5-FU c.i.)	cT3<br>ECOG PS ≤ 2<br>NEU >1.5x109/L<br>PLT >100X109/L<br>bilirubin and ALT <1.5 x ULN<br>creatinine < 1.5 x ULN	2001- 2006	323	153	ypT0:<br>SC-RT 1%<br>NAD-CRT 15%	LR, DM, OS	- no significant difference in outcomes;<br>- NAD-CRT may be more effective in reducing LR for distal tumors.
ACCORD 12/0405 PRODIGE 2 Gerard GP [19] (2010)	NAD-CRT (CAP45: RT 45Gy + Cap) Vs NAD-CRT (CAPOX50: RT 50Gy + Cap and Oxa)	T3-4 Nx M0<br>T2 Nx distal anterior rectum	2005-2008	598	524	CAP45 13.9%<br>CAPOX50 19.2%	pCR	Intensified NAD-CRT (CAPOX50):<br>- brings no significant difference on outcome;<br>- is not recommended.
I-CNR-RT, Sainato A [20] (2014)	NAD-CRT (45 Gy + 5-FU first and last week) followed by follow-up (group A) vs ADJ-CT with 5-FU (group B)	cT3-T4<br>Tumor located within 15 cm from anal margin<br>Age <75<br>ECOG PS <2<br>WBC >3000/L<br>Plt >130000/L<br>Creatinine <1.2 mmol/L<br>No previous history of cancer	1992-2001	634	476	Group A 17%<br>Group B 18.6%	OS	Addition of ADJ-CT does not improve OS and DFS

Chronicle,<br>Glynne-Jones R [21]<br>(2014)	NAD-CRT with fluoropyrimidine (45 Gy) followed by follow-up (Observation arm)<br>Vs ADJ-CT (XELOX arm, 6 cycles Cap + Oxa)	Tumor located within 15 cm from anal margin or below peritoneal reflection<br>Age >18<br>ECOG PS <2<br>Adequate hematologic, hepatic, renal function<br>M0<br>R0<br>CRM >1 mm	2004-2008	113	91	-	DFS	The study was terminated early (poor accrual). No significant difference in DFS or OS was found for adjuvant XELOX.
CAO/ARO/AIO-04,<br>Rödel C [13]<br>(2015)	NAD-CRT (50Gy + 5-FU first and last week) + ADJ-CT (4 cycles 5-FU)<br>Vs NAD-CRT (50Gy + 5-FU first and last week + Oxa days 1, 8, 22, 29) + ADJ-CT (8 cycles 5-FU + Oxa)	cT3-4, N1-2<br>Age >18<br>ECOG PS ≤ 2<br>Hb >10 g/dL<br>NEU >1.500 cells/μL<br>PLT >100000 cells/μL<br>Total bilirubin <2mg/dL<br>AST, ALT, alkaline phosphatase, γ-GT <3 × ULN<br>creatinine <1,5 mg/dL<br>calculated creatinine clearance <50mL/min	2006- 2010	1236	1008	5-FU group 13%<br>5-FU + Oxa group 17%	DFS	Adding Oxa to 5FU-based NAD-CRT and ADJ-CT significantly improved DFS.
Polish II,<br>Bujko K [15]<br>(2016)	Group A SC-RT (25Gy) + consolidation CT (3 cycles 5-FU+Oxa)<br>Vs group B NAD-CRT (50.4 Gy + 5-FU first and last week + Oxa days 1, 8, 15, 22, 29)	Fixed cT3 or cT4 tumors<br>Age <75<br>ECOG PS ≤2	2008-2014	515	157	Group A 16%<br>Group B 12%	R0 resection rate	- no difference in local efficacy between the two arms<br>- improved OS and lower acute toxicity for SC-RT + consolidation CT
INTERACT,<br>Valentini V [22]<br>(2019)	NAD-CRT (XELOX-RT: 50.4Gy + Cap 1300 mg/m2 + Oxa days 1, 19 and 38)<br>Vs NAD-CRT (XELAC-RT: 55Gy + Cap 1650 mg/m2)	Age >18<br>cT2N0-2 low located tumor, cT3 N0-2<br>Resectable disease<br>KPS >60<br>WBC >4.000 cells/ml<br>PLT > 100.000 cells/ml	2005-2014	534	304	XELAX 24.4%<br>XELOX 23.8%	Pathological major downstaging (TRG1-TRG2)	- XELOX-RT causes higher toxicity;<br>- no differences in terms of TRG distribution.

pCR: pathological complete response; NAD-CRT: neoadjuvant chemo-radiotherapy; 5FU: 5-fluorouracil; ADJ-CRT: adjuvant chemo-radiotherapy; LC: local control; OS: overall survival; SC-RT: short course radiotherapy; ADJ-CT: adjuvant chemotherapy; DRE: digital rectal examination; DFS: disease free survival; LR: local recurrence; nRT: neoadjuvant radiotherapy; ECOG PS: Eastern Cooperative Oncology Group Performance Status; c.i.: continuous infusion; NEU: neutrophils; PLT: platelets; ALT: Alanine Aminotransferase; ULN: upper limit of normal; Cap: capecitabine; Oxa: oxaliplatin; WBC: White Blood Cells; CRM: circumferential resection margin; Hb: haemoglobin; AST: Aspartate Aminotransferase; γ-GT: glutamyltransferase; KPS: Karnofsky Performance Status; TRG: Tumor Residual Grade.

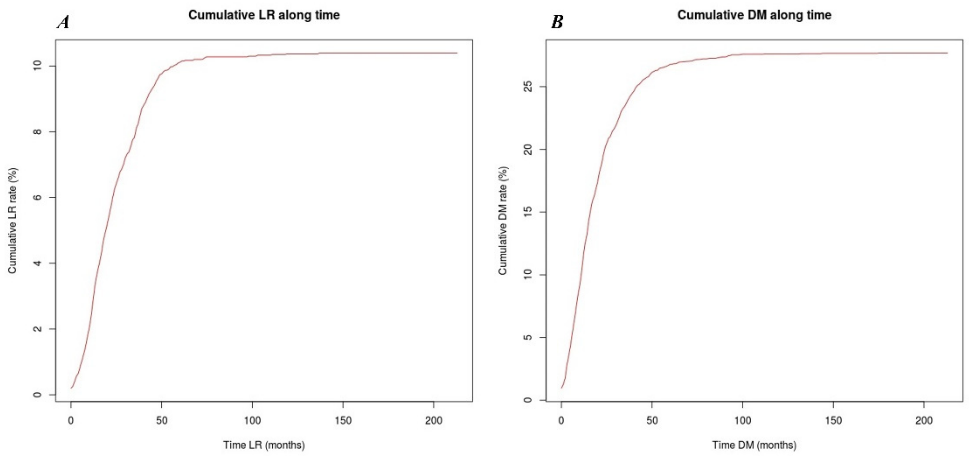

Figure S2. Cumulative rates for local recurrence (A) and distant metastasis (B) for the pooled database.

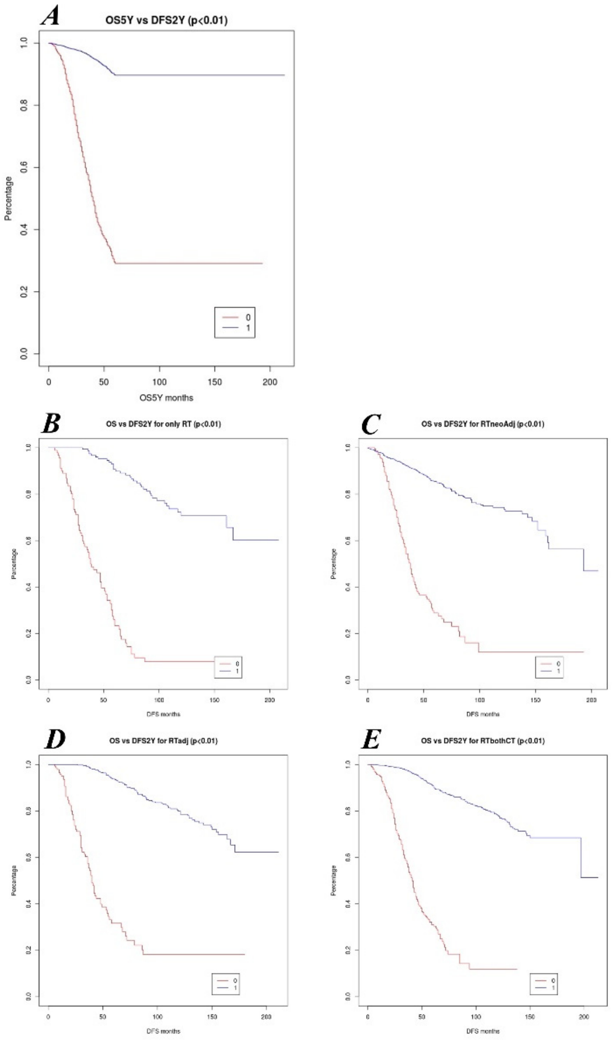

Figure S2. Overall survival plots stratified for 2yDFS status for the whole population (A) and the treatment subgroups of nRT, nCRT, nRT-adjCT and nCRT-adjCT (B–E) respectively.

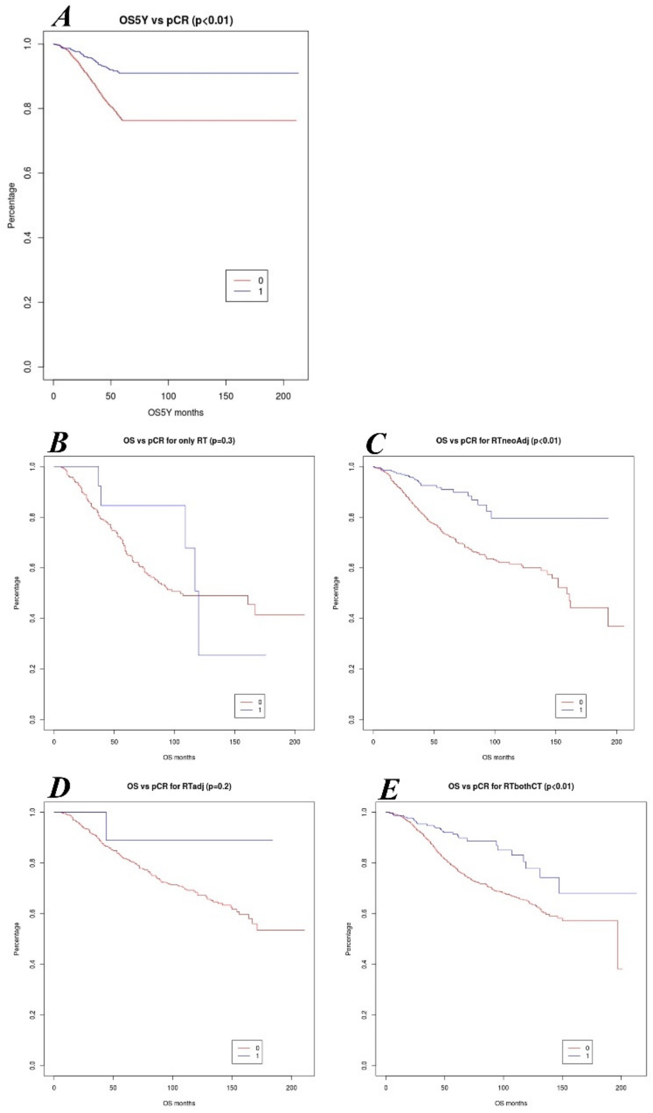

Figure S3. Overall survival plots stratified for pCR status for the whole population (A) and the treatment subgroups of nRT, nCRT, nRT-adjCT and nCRT-adjCT (B–E) respectively.

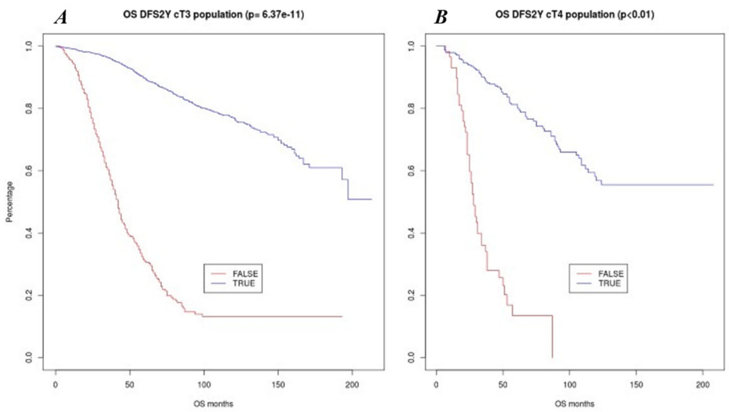

Figure S4. Overall survival plots stratified for 2yDFS status in cT3 (A) and cT4 (B) patients.

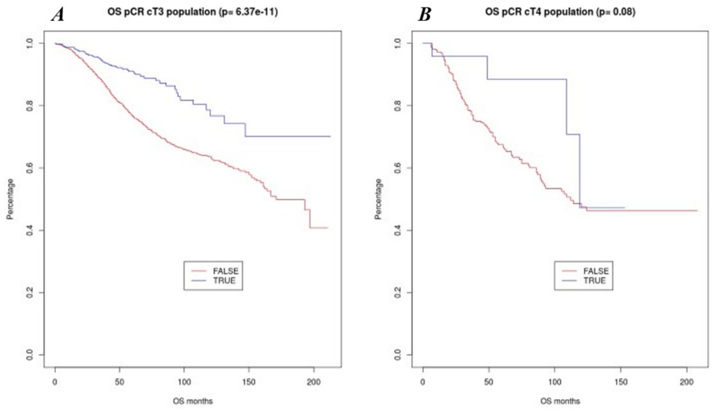

Figure S5. Overall survival plots stratified for pCR status in cT3 (A) and cT4 (B) patients.

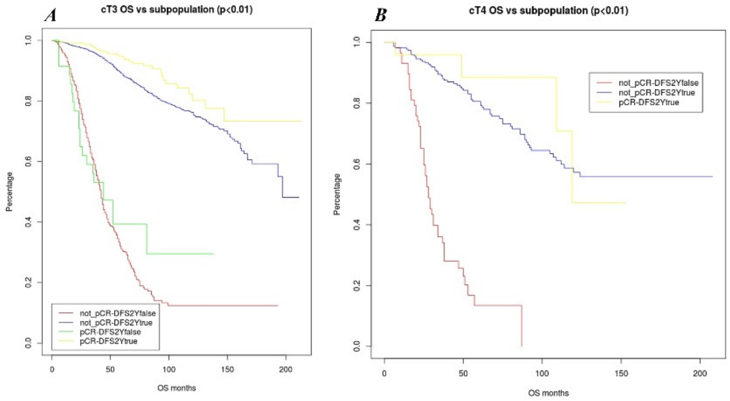

Figure S6. Overall survival plots stratified for combined status of pCR and 2yDFS in cT3 (A) and cT4 (B) patients.

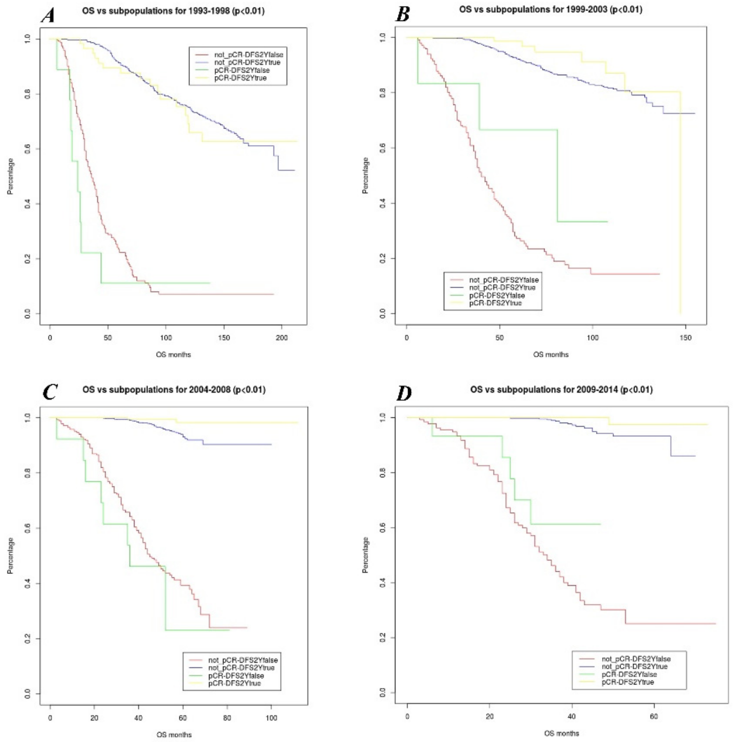

Figure S7. Overall survival plots stratified for combined status of pCR and 2yDFS according to accrual time (A–D).
